# Evaluation of Proteoforms of the Transmembrane Chemokines CXCL16 and CX3CL1, Their Receptors, and Their Processing Metalloproteinases ADAM10 and ADAM17 in Proliferative Diabetic Retinopathy

**DOI:** 10.3389/fimmu.2020.601639

**Published:** 2021-01-20

**Authors:** Ahmed M. Abu El-Asrar, Mohd Imtiaz Nawaz, Ajmal Ahmad, Alexandra De Zutter, Mohammad Mairaj Siddiquei, Marfa Blanter, Eef Allegaert, Priscilla W. Gikandi, Gert De Hertogh, Jo Van Damme, Ghislain Opdenakker, Sofie Struyf

**Affiliations:** ^1^ Department of Ophthalmology, College of Medicine, King Saud University, Riyadh, Saudi Arabia; ^2^ Dr. Nasser Al-Rashid Research Chair in Ophthalmology, College of Medicine, King Saud University, Riyadh, Saudi Arabia; ^3^ Laboratory of Molecular Immunology, Department of Microbiology, Immunology and Transplantation, Rega Institute for Medical Research, University of Leuven, Leuven, Belgium; ^4^ Laboratory of Histochemistry and Cytochemistry, University of Leuven, Leuven, Belgium; ^5^ Laboratory of Immunobiology, Department of Microbiology, Immunology and Transplantation, Rega Institute for Medical Research, University of Leuven, Leuven, Belgium

**Keywords:** proliferative diabetic retinopathy, chemokines, metalloproteinases, CXCL16, CX3CL1, ADAM10, ADAM17

## Abstract

The transmembrane chemokine pathways CXCL16/CXCR6 and CX3CL1/CX3CR1 are strongly implicated in inflammation and angiogenesis. We investigated the involvement of these chemokine pathways and their processing metalloproteinases ADAM10 and ADAM17 in the pathophysiology of proliferative diabetic retinopathy (PDR). Vitreous samples from 32 PDR and 24 non-diabetic patients, epiretinal membranes from 18 patients with PDR, rat retinas, human retinal Müller glial cells and human retinal microvascular endothelial cells (HRMECs) were studied by enzyme-linked immunosorbent assay, immunohistochemistry and Western blot analysis. *In vitro* angiogenesis assays were performed and the adherence of leukocytes to CXCL16-stimulated HRMECs was assessed. CXCL16, CX3CL1, ADAM10, ADAM17 and vascular endothelial growth factor (VEGF) levels were significantly increased in vitreous samples from PDR patients. The levels of CXCL16 were 417-fold higher than those of CX3CL1 in PDR vitreous samples. Significant positive correlations were found between the levels of VEGF and the levels of CXCL16, CX3CL1, ADAM10 and ADAM17. Significant positive correlations were detected between the numbers of blood vessels expressing CD31, reflecting the angiogenic activity of PDR epiretinal membranes, and the numbers of blood vessels and stromal cells expressing CXCL16, CXCR6, ADAM10 and ADAM17. CXCL16 induced upregulation of phospho-ERK1/2, p65 subunit of NF-κB and VEGF in cultured Müller cells and tumor necrosis factor-α induced upregulation of soluble CXCL16 and ADAM17 in Müller cells. Treatment of HRMECs with CXCL16 resulted in increased expression of intercellular adhesion molecule-1 (ICAM-1) and increased leukocyte adhesion to HRMECs. CXCL16 induced HRMEC proliferation, formation of sprouts from HRMEC spheroids and phosphorylation of ERK1/2. Intravitreal administration of CXCL16 in normal rats induced significant upregulation of the p65 subunit of NF-κB, VEGF and ICAM-1 in the retina. Our findings suggest that the chemokine axis CXCL16/CXCR6 and the processing metalloproteinases ADAM10 and ADAM17 might serve a role in the initiation and progression of PDR.

## Introduction

Ischemia-induced retinal angiogenesis, inflammation and fibrosis are the pathological hallmarks of proliferative diabetic retinopathy (PDR) and are critical mechanisms for PDR initiation and progression. The surgically excised epiretinal fibrovascular membranes from patients with PDR are characterized by the presence of new blood vessels in addition to leukocytes and α-smooth muscle actin (α-SMA)-expressing myofibroblasts in the stromal compartment (1–5). Myofibroblasts are the key cellular mediators of fibrosis and their presence is a marker of progressive fibrosis (6). In addition, several studies demonstrated the overexpression of inflammatory, angiogenic and fibrogenic factors in the ocular microenvironment of patients with PDR (1–5, 7).

Hypoxia seems to be a critical inducer for neovascularization in PDR by upregulating the production of angiogenic factors (8, 9). Research in the field of diabetic retinopathy led to the identification of the proangiogenic factor vascular endothelial growth factor (VEGF) as an important mediator of PDR angiogenesis (10, 11). Hypoxia-mediated induction of VEGF has been demonstrated in retinal cells (12). In addition to angiogenesis, recruitment of leukocytes occurs in the ocular microenvironment of patients with PDR (1–5). Increased expression of the adhesion molecule intercellular adhesion molecule-1 (ICAM-1), retinal leukocyte stasis and enhanced adhesion of leukocytes to the retinal microvascular endothelium play central roles in the development of diabetic endothelial cell damage, breakdown of the blood-retinal barrier and capillary nonperfusion (13). Increasing evidence supports the causal relationship between persistent inflammation and angiogenesis (14, 15). These observations suggest that the link between inflammation and angiogenesis plays critical roles in PDR initiation and progression. This paradigm is supported by the dysregulated expression of multiple signaling molecules of the inflammatory response with angiogenic activities, such as chemokines (4, 16–20) and matrix metalloproteinases (2, 21) in the ocular microenvironment of patients with PDR. Therefore, molecules with roles in inflammation and pathological neovascularization are considered potential targets for treatment of PDR.

Chemokines are a superfamily of 8- to 10-kDa multifunctional mediators that direct the recruitment of leukocytes to sites of inflammation, promote inflammation and enhance immune responses. They are divided into four subgroups, CXC, CC, C and CX3C, depending on the arrangement of the conserved cysteine residues. The specific effects of chemokines are mediated by binding to distinct members of a family of G protein-coupled receptors (22). Although chemokines are generally thought to function as leukocyte attractants, there is increasing evidence that multiple chemokines and their corresponding receptors play critical roles in regulating angiogenesis (23–25).

Two chemokines are unique members of the chemokine family, because they are considerably larger (more than 20-kDa) and because they are anchored into membranes. Indeed, CXCL16, also named scavenger receptor for phosphatidyl serine and oxidized low-density lipoprotein (SR-PSOX), and fractalkine/CX3CL1 are synthesized as transmembrane molecules that can be cleaved from the cell surface to produce soluble forms. When expressed at the surface of endothelial cells as transmembrane molecules, CXCL16 and CX3CL1 function as adhesion molecules and interact with their cognate receptors CXCR6 and CX3CR1, respectively, which are expressed on leukocyte subtypes. Soluble forms are generated by proteolytic release from the cell surface, a process which is called ectodomain shedding. Proteolytic cleavage of the transmembrane chemokines appears to be essential for their switch from a mainly adhesive to a potent leukocyte chemoattractive function similar to other members of the chemokine family (22). The homeostatic release of both CXCL16 and CX3CL1 is mainly mediated by the disintegrin and metalloproteinase (ADAM) 10 (26–29), whereas ADAM17, also referred to as tumor necrosis factor-alpha converting enzyme (TACE), was identified as the protease responsible for inducible shedding of CXCL16 and CX3CL1 (28, 30, 31). Thus, the activities of ADAM10 and ADAM17 may be regarded as prerequisite for the proangiogenic and proinflammatory effects of CXCL16 and CX3CL1.

Accumulated evidence points toward a role of the chemokine pathways CXCL16/CXCR6 (32–34) and CX3CL1/CX3CR1 (35–37) in autoimmune and inflammatory diseases. In addition to their roles in leukocyte recruitment and inflammation, several *in vitro* and *in vivo* studies demonstrated that CXCL16 (38–42) and CX3CL1 (43–46) stimulate the angiogenic process. These data indicate that CXCL16 and CX3CL1 are inflammatory chemokines with angiogenic activities and are mediators of inflammatory angiogenesis. Given the key roles of CXCL16 and CX3CL1 in inflammation and angiogenesis, we investigated the involvement of these chemokines and their processing metalloproteinases ADAM10 and ADAM17 in the pathophysiology of PDR.

## Materials and Methods

### Patients Samples

Undiluted vitreous fluid samples were obtained from 32 patients with PDR during pars plana vitrectomy for the treatment of tractional retinal detachment, and/or nonclearing vitreous hemorrhage and processed as described previously (2–5, 7). Vitreous fluid samples obtained from 24 patients who had undergone vitrectomy for the treatment of rhegmatogenous retinal detachment with no proliferative vitreoretinopathy (PVR) were used as the control samples. Clinical check-up confirmed that control subjects were free from diabetes or other systemic disease. Epiretinal fibrovascular membranes were obtained from 18 patients with PDR undergoing pars plana vitrectomy for the repair of tractional retinal detachment. At the time of the procedure, using previously published criteria, retinal neovascular activity was clinically graded (47) into active neovascularization (visible perfused new vessels on the retina or optic disc present within epiretinal membranes) and inactive involuted disease (nonvascularized, white fibrotic epiretinal membranes). For comparison, epiretinal fibrocellular membranes were obtained from 10 patients without diabetes undergoing vitreoretinal surgery for the treatment of retinal detachment complicated by PVR. The epiretinal membranes were processed as previously described (1–3, 5, 7). Immunohistochemical staining was performed on two-micrometer thick formalin-fixed, paraffin embedded sections.

The study was conducted according to the tenets of the Declaration of Helsinki. Before undergoing vitrectomy, all patients signed a preoperative informed written consent and approved the use of the excised epiretinal membranes and aspirated vitreous fluid for further analysis and clinical research. The Research Centre and Institutional Review Board of the College of Medicine, King Saud University, approved the study design and protocol.

### Immunohistochemical Staining of Human Epiretinal Membranes and Quantitations

For CD31, α-SMA, CX3CL1, CX3CR1 and ADAM10 detection, antigen retrieval was performed by boiling the sections in citrate-based buffer [pH 5.9 – 6.1] [BOND Epitope Retrieval Solution 1; Leica, Diegem, Belgium] for 10 min. For CD45, CXCL16, CXCR6 and ADAM17 detection, antigen retrieval was performed by boiling the sections in Tris/EDTA buffer [pH 9] [BOND Epitope Retrieval Solution 2; Leica] for 20 min. Subsequently, the sections were incubated for 1 h with mouse monoclonal anti-CD31 (ready-to-use; clone JC70A; Dako, Glostrup, Denmark), mouse monoclonal anti-CD45 (ready-to-use; clones 2B11+PD7/26; Dako), mouse monoclonal antibody against α-SMA (ready-to-use; clone 1A4; Dako), rabbit polyclonal anti-CXCL16 antibody (1:400; LS-B8223, Lifespan Bioscience, Inc., Seattle, WA, USA), rabbit polyclonal anti-CXCR6 antibody (1:50; ab137344, Abcam, Cambridge, UK), rabbit polyclonal anti-CX3CL1 antibody (1:200; ab9819, Abcam), mouse monoclonal anti-CX3CR1 antibody (1:50; ab184678, Abcam), rabbit polyclonal anti-ADAM10 antibody (1:100; ab39177, Abcam) and rabbit polyclonal anti-ADAM17 antibody (1:200; ab39162, Abcam). Optimal working conditions for the antibodies were determined in pilot experiments on lung, kidney, tonsil, spleen and liver sections. The sections were then incubated for 20 min with an alkaline phosphatase-conjugated IgG. Immune interactions were visualized with the Fast Red chromogen (Leica; 15 min incubation). Finally, a faint counterstain with Mayer’s hematoxylin was performed, when indicated.

To identify the phenotype of cells expressing CXCL16, CXCR6, CX3CL1, CX3CR1, ADAM10 and ADAM17, sequential double immunohistochemistry was performed. The sections were first incubated with anti-CD45, followed by treatment with peroxidase-conjugated secondary antibody and 3, 3’-diaminobenzidine tetrahydrochloride substrate. Next, the antibodies (anti-CXCL16, anti-CXCR6, anti-CX3CL1, anti-CX3CR1, anti-ADAM10 or anti-ADAM17) were added and detected by alkaline phosphatase-conjugated secondary antibody and Fast Red reactions. No counterstain was applied. In negative controls, the incubation step with primary antibody was omitted from the protocol and only the ready-to-use antibody diluent (Cat No 52022; Dako) was applied.

To visualize the angiogenic process and to determine the level of vascularization in epiretinal membranes, immunodetection of the vascular endothelium marker CD31 was performed. Immunoreactive blood vessels and cells were counted in five representative fields, with the use of an eyepiece with calibrated grid in combination with the 40x objective as previously described (3, 5, 7).

### Enzyme-Linked Immunosorbent Assays

Enzyme-linked immunosorbent assay (ELISA) kits for human CXCL16 (Cat No DCX160), human ADAM17 (Cat No DY930) and human VEGF (Cat No SVE00) were purchased from R&D Systems (Minneapolis, MN, USA). The ELISA kit for human CX3CL1 (Cat No ab192145) was purchased from Abcam and the human ADAM10 ELISA (Cat No abx492003) from Abbexa (Cambridge, UK). Levels of human CXCL16, ADAM17, VEGF, CX3CL1 and ADAM10 in vitreous fluid and CXCL16, CX3CL1 and VEGF in culture medium were determined using the aforementioned ELISA kits according to the manufacturer’s instructions. The minimum detection limits for CXCL16, VEGF, CX3CL1 and ADAM10 ELISA kits were 7 pg/ml, 9 pg/ml, 1.18 pg/ml and 28 pg/ml, respectively.

### Human Retinal Müller Glial Cell and Retinal Microvascular Endothelial Cell Culture

Human retinal Müller glial cells (MIO-M1) (a generous gift from Prof. A. Limb, Institute of Ophthalmology, University College London, UK) were cultured with DMEM containing 1 g/L glucose with 10% (v/v) fetal bovine serum and 1% penicillin/streptomycin. Confluent cultures were starved overnight in serum-free DMEM to minimize the effects of serum and subsequently either left untreated or stimulated for 24 h. The following stimuli were used: 1 or 10 ng/ml recombinant human CXCL16 (Cat No ab50175, Abcam), 300 μM of the hypoxia mimetic agent cobalt chloride (CoCl_2_) (AVONCHEM Limited, Nacclesfield, Cheshire, UK), or 50 ng/ml recombinant human tumor necrosis factor-alpha (TNF-α) (Cat No 210-TA, R&D Systems).

Human retinal microvascular endothelial cells (HRMECs) were purchased from Cell Systems Corporation (Kirkland, WA, USA) and maintained in complete serum-free media (Cat No SF-4Z0–500, Cell System Corporation) supplemented with “Rocket Fuel” (Cat No SF-4Z0–500, Cell System Corporation), “Culture Boost” (Cat No 4CB-500, Cell System Corporation), and antibiotics (Cat No 4Z0–643, Cell System Corporation) at 37°C in a humidified atmosphere with 5% CO_2_. We used HRMECs up to passage 8 for all the experiments. About 80% confluent cells were starved in a minimal medium (medium supplemented with 0.25% “Rocket Fuel” and antibiotics) overnight to eliminate any residual effects of growth factors. The following stimuli were used: 10 or 50 ng/ml recombinant soluble human CXCL16 (Cat No ab50175, Abcam), 50 ng/ml recombinant human TNF-α (Cat No 210-TA) or 50 ng/ml recombinant human interleukin-1 beta (IL-1β) (Cat No 201-LB) from R&D Systems. Cells were harvested after 24 h and lysed in radioimmunoprecipitation assay (RIPA) lysis buffer (sc-24948, Santa Cruz Biotechnology, Inc, Santa Cruz, CA, USA) for Western blot analysis.

### Intravitreal Injection of CXCL16

All procedures with animals were performed in accordance with the Association for Research in Vision and Ophthalmology (ARVO) statement for use of animals in ophthalmic and vision research and were approved by the institutional animal care and use committee of the College of Pharmacy, King Saud University. Sprague Dawley rats (220-250 g) were kept under deep anesthesia, and sterile recombinant human CXCL16 (5 ng/5 μl; Cat No ab50175, Abcam) was injected into the vitreous of the right eye as previously described (7). As a control, the left eye received a sham injection of 5 μl of sterile phosphate-buffered saline (PBS). Animals were sacrificed one or 4 days after intravitreal administration. Subsequently, the retinas were carefully dissected, snap frozen in liquid nitrogen, and stored at -80°C until analysis.

### Western Blot Analysis of Human Vitreous Fluid, Rat Retinas, and Retinal Microvascular Endothelial Cell and Müller Cell Lysates

Retinas and cell lysates were homogenized in Western blot lysis buffer [30 mM Tris-HCl; pH 7.5, 5mM EDTA, 1% Triton X-100, 250 mM sucrose, 1 mM sodium vanadate, and a complete protease inhibitor cocktail from Roche (Mannheim, Germany)]. After centrifugation of the homogenates (14,000 × g; 15 min, 4°C), protein concentrations were measured in the supernatants (DC protein assay kit; Bio-Rad Laboratories, Hercules, CA). Equal amounts (50 μg) of the protein extracts were subjected to SDS–PAGE and transferred onto nitrocellulose membranes. To determine the presence of soluble CXCL16, ADAM10 and ADAM17 in the vitreous samples, equal volumes (15 μl) of vitreous samples were boiled in Laemmli’s sample buffer (1:1, v/v) under reducing conditions for 10 min and analyzed as described (2, 3, 5, 7).

Immunodetection was performed with the use of rabbit polyclonal anti-CXCL16 antibody (1:1000; ab101404, Abcam), rabbit polyclonal anti- CXCR6 antibody (1:1000; ab137344, Abcam), rabbit polyclonal anti-CX3CL1 antibody (1:1000, ab9819, Abcam), mouse monoclonal anti-CX3CR1 antibody (1:1000, ab184678, Abcam), mouse monoclonal anti-p65 subunit of nuclear factor-kappa B (NF-κB) antibody (1:500, sc-136548, Santa Cruz Biotechnology Inc.), rabbit monoclonal anti-phospho-extracellular signal-regulated kinase (ERK)1/2 antibody (1:1500, MAB1018, R&D Systems), rabbit polyclonal anti-ADAM10 antibody (1:1000, ab39177, Abcam), rabbit polyconal anti-ADAM17 antibody (1:1000, ab39163, Abcam), mouse monoclonal anti-ICAM-1 antibody (1:500, sc-8439, Santa Cruz Biotechnology Inc.) and mouse monoclonal anti-VEGF antibody (1:750, MAB293, R&D Systems). Membranes were stripped and reprobed with an antibody against β-actin (1:3000, sc-47778, Santa Cruz Biotechnology Inc.) to evaluate the sample processing and loading. Bands were visualized with the use of high-performance chemiluminescence (G: Box Chemi-XX8 from Syngene, Synoptic Ltd. Cambridge, UK) and the intensities were quantified by using GeneTools software (Syngene by Synoptic Ltd.).

### Cell Adhesion Assay

To determine leukocyte adhesion to stimulated HRMEC monolayers, we used the CytoSelect Leukocyte-endothelium adhesion kit (Cat No CBA-210, Cell Biolabs, Inc.). The assay protocol was followed as described previously (48). Briefly, 2x10^5^ HRMECs were seeded on 0.2% (v/v) gelatin-coated 24-well plates. After reaching a confluent monolayer, endothelial cells (starved overnight) were treated either with 10 or 50 ng/ml recombinant human CXCL16 (Cat No ab50175, Abcam) or with 50 ng/ml recombinant human TNF-α as positive control (Cat No 210-TA, R&D Systems) for 24 h. Next, 5x10^5^ monocytic THP-1 cell (American Type Culture Collection, Manassas, VA, USA) labeled with fluorescent LeukoTracker were added on top of treated HRMECs monolayer for 30 min. After washing, the remaining adherent THP-1 cells were lysed and the fluorescence was measured using a spectraMax Gemini-XPS (Molecular Devices, CA, USA) with excitation and emission wavelengths of 485 nm and 538 nm, respectively.

### Proliferation Assay

To examine the effect of CXCL16 on endothelial cell proliferation, 5x10^3^ HRMECs were seeded in a 96-well plate in 100 µl culture medium [Endothelial Cell Basal Medium-2 (EBM-2) supplemented with the SingleQuots kit; both from Lonza]. The next day, cells were washed with serum-free MCDB131 medium and stimulated in MCDB131 medium supplemented with 2mM GlutaMAX™, 30 µg/ml Gentamicin and 3% (v/v) FCS with different concentrations of human CXCL16 (R&D Systems) or 10 ng/ml human VEGF (Biolegend, San Diego, CA, USA) as a positive control. After 48 h, cell proliferation was assessed using the ATPlite Luminescence Assay kit (Perkin Elmer, Waltham, MA) according to the manufacturer’s instructions.

### Phospho-ERK1/2 Signal Transduction Assay

Activation of the ERK signaling pathway in HRMECs was assessed by measuring phosphorylation of ERK1/2. HRMECs were seeded in 6-well plates in culture medium (*vide supra*). Once the cultures reached 70-80% confluency, the cells were serum-starved overnight in MCDB131 medium. Fifteen min prior to stimulation, the cells were incubated in 900 µl MCDB131 medium supplemented with 0.5% (v/v) bovine serum albumin (BSA) at 37°C. Cells were stimulated with 100 µl of ten-times concentrated stimulus in the aforementioned medium or medium alone for 15 min at 37°C. Then, the cells were placed on ice, washed with ice-cold PBS and 90 µl of lysis buffer [1% (v/v) protease inhibitor cocktail, phosphatase inhibitor cocktail 2 and 3 (all Sigma Aldrich) in 1 mM EDTA, 0.5% (v/v) Triton X-100, 5 mM NaF, 6 M ureum] was added per well. After a 15-minute incubation on ice, the cell lysates were collected using a cell scraper and left on ice for another 5 min. The cell lysates were centrifuged at 550 g at 4°C for 5 min and the samples were stored at -20°C until analysis. The amount of phospho-ERK1/2 in the cell lysates was determined using an ERK1 (Thr202/Tyr204)/ERK2 (Thr185/Tyr187) ELISA duoset (R&D Systems) according to the manufacturer’s instructions.

### Spheroid Sprouting Assay

A spheroid sprouting assay was performed to verify the angiogenic effect of CXCL16. Cell suspensions of 1,000 HRMECs in 25 µl of culture medium with 20% methyl cellulose (Sigma-Aldrich) were plated on a petri dish and allowed to form single spheroids from a hanging droplet at 37°C, 5% CO_2_ for 24** h**. The spheroids were sedimented and embedded in a collagen-based matrix in a 96-well plate. They were overlaid with 45% methyl cellulose, 40% rat tail type I collagen (1.5 mg/ml; Ibidi), 15% NaHCO_3_ (2.5 mg/ml) and 1% NaOH (10 mM). Dilutions of CXCL16 in EBM-2 + 3% (v/v) FCS were added to obtain a final concentration of 0.3 and 3 ng/ml of CXCL16. After 14** h** spheroid sprouting was assessed with an inverted microscope (10x magnification; Zeiss) by counting the number of sprouts originating from a spheroid.

### Statistical Analysis

Statistical analyses of the data were performed using SPSS version 21.0. Normal distribution of the data was verified using the Shapiro-Wilk (S-W) test and normal Q-Q plots. Normally distributed data were presented as mean ± SD and One-Way ANOVA and independent t-test were used to compare the groups. For normally distributed data, Pearson correlation coefficients were calculated. Non-parametric tests (Kruskal-Wallis test, Mann-Whitney test and Spearman’s correlation coefficients) were performed for not normally distributed data, which were presented as median and interquartile range (IQR; Q1-Q3). The paired samples t-test and Wilcoxon test were used to compare the levels of the chemokines and ADAMs for normally and non-normally distributed data, respectively. The level of statistical significance was set at 0.05.

## Results

### Comparisons of Transmembrane Chemokines, Processing ADAMs and VEGF Levels in Vitreous Samples From Patients With PDR and Non-diabetic Control Patients

CXCL16, CX3CL1, ADAM10 and ADAM17 were detected in all vitreous samples. CXCL16 and CX3CL1 levels in vitreous samples from PDR patients were significantly higher than the levels in non-diabetic controls (p<0.001 for both comparisons; Mann-Whitney test) (**Table 1**). Similarly, ADAM10 and ADAM17 levels in PDR patients were significantly higher than the levels in non-diabetic controls (p<0.001; p=0.005, respectively; Mann-Whitney test). VEGF was detected in 16 of 24 (66.6%) vitreous samples from non-diabetic control patients and in all vitreous samples from patients with PDR. VEGF levels in vitreous samples from patients with PDR were significantly higher than the levels in non-diabetic control patients (p<0.001; Mann-Whitney test) (**Table 1**).

**Table 1:** Comparisons of vascular endothelial growth factor (VEGF), CXCL16, CX3CL1, ADAM10 and ADAM17 levels in proliferative diabetic retinopathy (PDR) patients with or without active neovascularization and nondiabetic patients with rhegmatogenous retinal detachment (RD).

Disease group	VEGF (pg/ml)	CXCL16 (pg/ml)	CX3CL1 (pg/ml)	ADAM10 (pg/ml)	ADAM17 (pg/ml)
• RD (n=24) Median [IQR]	11.0 (0.0–46.8)	2156.3 (1456.3–2903.1)	4.7 (1.3–7.4)	1215.0 (1108.3–1409.2	315.6 190.8–343.6)
• All PDR (n=32) Median [IQR]	185.5 (92.3–1484.5)	9306.3 (6543.8–9881.3)	22.3 (15.3–25.0)	1870.0 (1569.2–2217.5)	486.1 (226.9–1365.8)
p-value (Mann-Whitney test)	<0.001*	<0.001*	<0.001*	<0.001*	0.005*
• Active PDR (n=16) Median [IQR]	1424.5 (103.3–1657.5)	9593.8 (9321.9–9890.6)	23.7 (21.8–38.2)	2128.3 (1817.5–2585.0)	1433.3 (1092.5–1783.9)
• Inactive PDR (n=16) Median [IQR]	108.0 (77.3–365.8)	6543.8 (5281.3–9706.3)	16.3 (11.6–23.7)	1671.7 (1335.0–2050.0)	237.8 (215.6–429.2)
• RD (n=24) Median [IQR]	11.0 (0.0–46.8)	2156.3 (1456.3–2903.1)	4.7 (1.3–7.4)	1215.0 (1108.3–1409.2	315.6 190.8–343.6)
p-value (Kruskal-Wallis test)	<0.001*	<0.001*	<0.001*	<0.001*	<0.001*

*Statistically significant at 5% level of significance.

IQR, interquartile range.

### ELISA Levels of Transmembrane Chemokines and Processing ADAMs in Vitreous Samples From Non-diabetic Control Patients and Patients With PDR

Within both patient groups, CXCL16 levels were significantly higher than CX3CL1 levels (p<0.001; Wilcoxon test) (**Table 1**). The levels of CXCL16 were 458.8-fold and 417.3-fold higher than those of CX3CL1 in non-diabetic controls and PDR patients, respectively. The levels of ADAM10 were significantly higher than ADAM17 levels (p<0.001; Wilcoxon test) (**Table 1**).

### Relationship Between ELISA Levels of Transmembrane Chemokines, Processing ADAMs and VEGF in Vitreous Samples and Angiogenic Activity of PDR

Comparisons of CXCL16, CX3CL1, ADAM10, ADAM17 and VEGF levels among PDR patients with active neovascularization (n=16), PDR patients with inactive involuted neovascularization (n=16), and non-diabetic control patients (n=24) was conducted with the Kruskal-Wallis test. The levels differed significantly between the 3 groups (p<0.001 for all comparisons) (**Table 1**). Pairwise comparisons (Mann-Whitney test) indicated that CXCL16, CX3CL1, ADAM10, ADAM17 and VEGF levels were significantly higher in patients with active PDR than in PDR patients with inactive involuted neovascularization (p=0.016; p=0.006; p=0.008; p<0.001; 0.007, respectively) and control patients (p<0.001 for all comparisons). In addition, CXCL16, CX3CL1, ADAM10 and VEGF levels in patients with inactive involuted PDR were significantly higher than the levels in control patients (p<0.001 for all comparisons). However, the levels of ADAM17 did not differ significantly between patients with inactive involuted PDR and control patients (p=0.825).

### Correlations Between Vitreous Fluid Levels of Transmembrane Chemokines, Processing ADAMs, and VEGF

Significant positive correlations (Spearman’s correlation coefficient) were found between vitreous fluid levels of CXCL16 and levels of ADAM10 (r=0.741; p<0.001) and ADAM17 (r=0.289; p=0.044). Significant positive correlations were observed between vitreous fluid levels of CX3CL1 and levels of ADAM10 (r=0.581; p<0.001) and ADAM17 (r=0.406; p=0.004). In addition, the correlations between levels of CXCL16 and levels of CX3CL1 (r=0.745; p<0.001) and between levels of ADAM10 and ADAM17 (r=0.353; p=0.012) were significant. Furthermore, VEGF levels correlated significantly with the levels of CXCL16 (r=0.673; p<0.001). CX3CL1 (r=0.800; p<0.001), ADAM10 (r=0.608; p<0.001) and ADAM17 (r=0.468; p<0.001).

### Western Blot Analysis of Vitreous Samples

**Figure 1:**
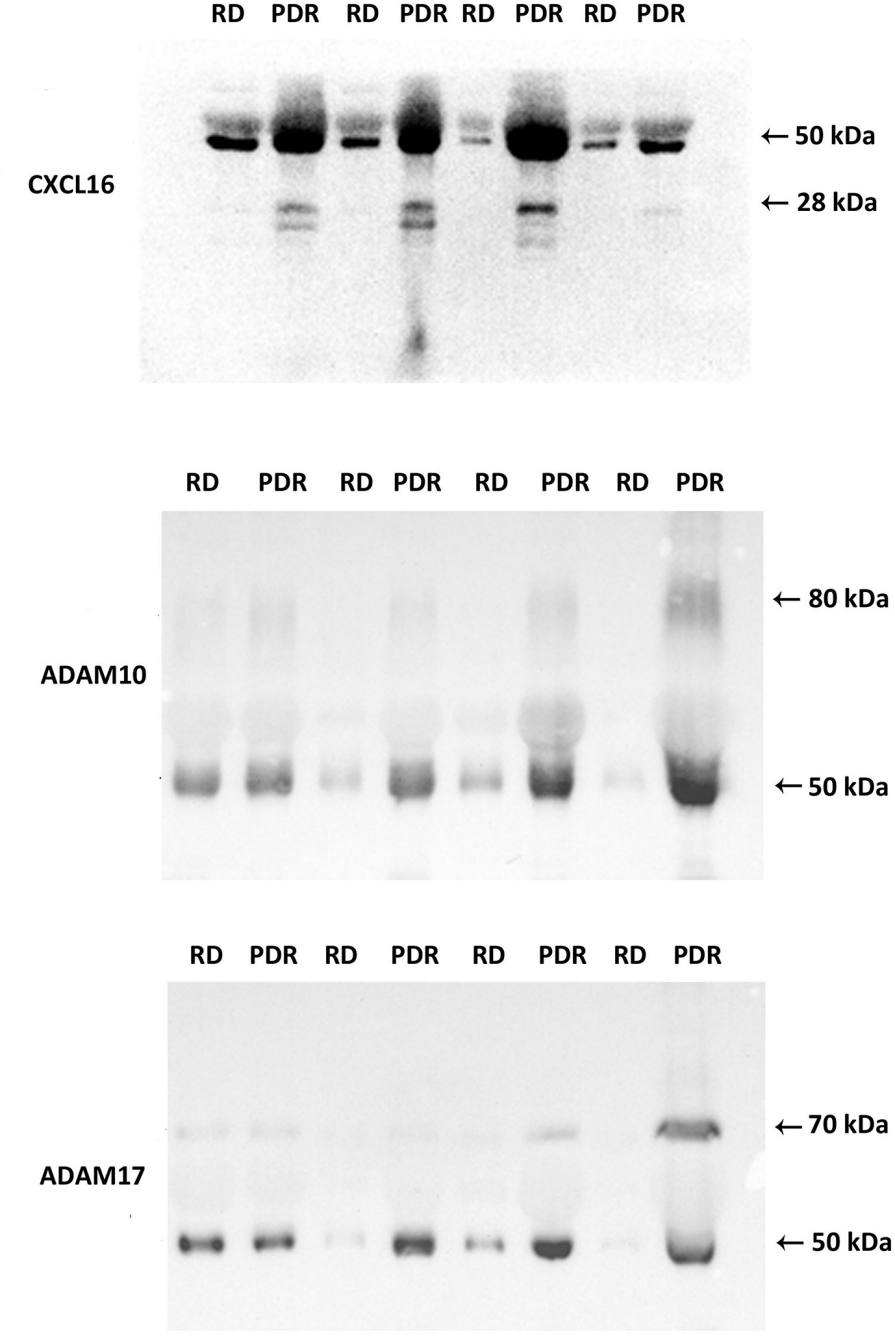
Detection of CXCL16, ADAM10, and ADAM17 in vitreous fluid. The expression of CXCL16, ADAM10, and ADAM17 in equal volumes (15 μl) of vitreous fluid samples from patients with proliferative diabetic retinopathy (PDR) (n=8) and from non-diabetic patients with rhegmatogenous retinal detachment (RD) (n=8) was determined by Western blot analysis. Immunoreactive proteoforms are indicated in kilodaltons (kDa) on the basis of a size standard preparation. Representative sets of samples are shown.

The transmembrane chemokines and ADAMs exist in various protein forms (proform, activated form, membrane-anchored form, soluble form and/or degradation products), henceforth called proteoforms. The ELISA data represent general numerical values of the immunoreactivity from the whole collection of proteoforms of a specific protein in a sample. For this reason, we added Western blot analysis as an extra dimension to provide insights into the relative abundancies of the various proteoforms of the individual molecules in this study. With the use of Western blot analysis of equal volumes of vitreous fluid, we confirmed the presence of CXCL16, ADAM10 and ADAM17 in vitreous samples (**Figure 1**). The relative levels in PDR patients (n=8) versus non-diabetic controls (n=8) were in line with the ELISA results (**Table 1**).

Western blot analysis of vitreous fluid samples, revealed that CXCL16 was expressed as two different forms: a 50 kDa doublet form and a 28 kDa doublet form. The basis for this is not known but has been speculated to be due to differences in the extent or type of glycosylation (49, 50). Most of the CXCL16 immunoreactivity appeared at the level of 50 kDa form (**Figure 1**). ADAM10 was expressed as two protein bands at 80 kDa and 50 kDa. ADAM17 was also expressed as two protein bands at 70 kDa and 50 kDa (**Figure 1**). These forms might represent the soluble ectodomains of ADAM10 and ADAM17. It was demonstrated that ADAM10 and ADAM17 can be proteolytically released from the cell surface and that the soluble forms exhibit additional biological roles (51).

### Neovessels, Leukocytes, and Myofibroblasts in Epiretinal Fibrovascular Membranes From Patients With PDR

**Figure 2:**
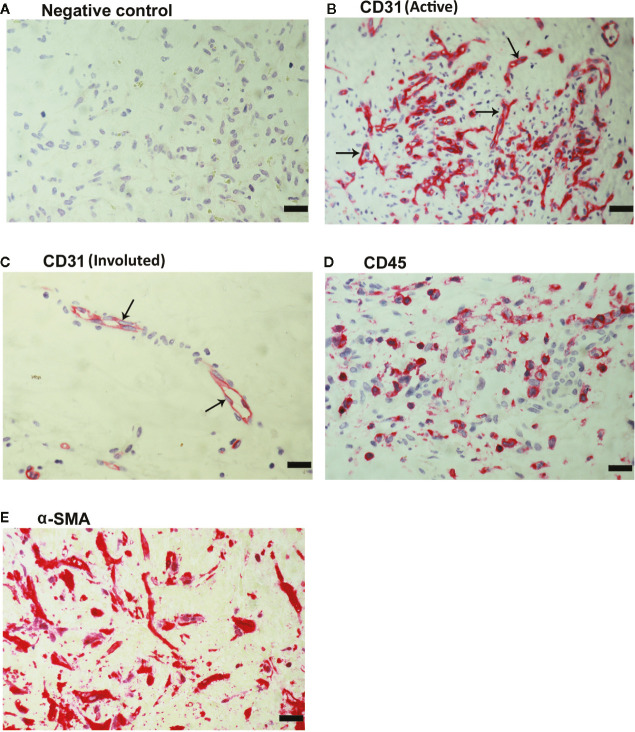
Detection of pathologic new blood vessels, leukocytes and myofibroblasts in proliferative diabetic retinopathy epiretinal fibrovascular membranes. No labeling was observed in the negative control slide (the same procedure without the primary antibody) **(A)**. Immunohistochemical staining for the endothelial cell marker CD31 showing pathologic new blood vessels in an epiretinal membrane from a patient with active neovascularization **(B)** and in a membrane from a patient with inactive involuted disease which is composed mostly of fibrous tissue (arrows) **(C)**. Immunohistochemical staining for the leukocyte common antigen CD45 showing infiltrating leukocytes in the stroma **(D)**. Immunohistochemical staining for α-smooth muscle actin (α-SMA) showing immunoreactivity in spindle-shaped myofibroblasts **(E)** (scale bar, 10 μm).

As a negative control, the immunohistochemical staining procedure was performed with omission of the primary antibody from the protocol. No staining was observed in the negative control slides (**Figure 2A**). Subsequently, we used staining for the vascular endothelial cell marker CD31 to determine the levels of vascularization in epiretinal fibrovascular membranes from patients with PDR. All membranes showed neovessels positive for this vascular endothelial cell marker. Representative immunohistochemical stainings for CD31 indicating new blood vessels in a membrane from a patient with active PDR (**Figure 2B**) and in a membrane from a patient with involuted PDR (**Figure 2C**) are shown. In addition, leukocytes expressing the leukocyte common antigen CD45 (**Figure 2D**), as well as spindle-shaped myofibroblasts expressing α-SMA (**Figure 2E**) were detected in the stromal compartment.

### Expression of the Transmembrane Chemokines and Their Receptors in Epiretinal Fibrovascular Membranes From Patients With PDR

**Figure 3:**
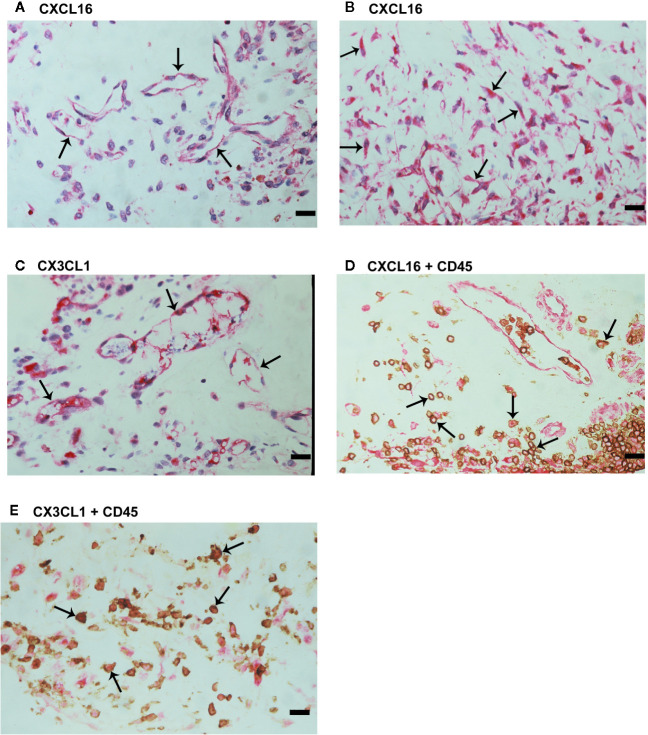
Characterization of cells expressing CXCL16 and CX3CL1 in proliferative diabetic retinopathy epiretinal fibrovascular membranes. Immunohistochemical staining for CXCL16 **(A)** and CX3CL1 **(C)** showing immunoreactivity in vascular endothelial cells (arrows). Immunoreactivity for CXCL16 was also detected in stromal spindle-shaped cells (arrows) **(B)**. Double immunohistochemistry for CD45 (brown) and CXCL16 (red) **(D)** or CX3CL1 (red) **(E)** demonstrated co-expression in stromal leukocytes (arrows). No counterstain was applied in panels **(D, E)** (scale bar, 10 μm).

We used immunohistochemical analysis to reinforce the understanding of the observed alterations of these analytes in the vitreous, to identify the cellular source of vitreous fluid transmembrane chemokines and to examine their tissue localization and expression in epiretinal fibrovascular membranes from 18 patients with PDR. CXCL16 (**Figures 3A, B**) and CX3CL1 (**Figure 3C**) immunoreactivities were observed in all membranes. Immunoreactivities for CXCL16 (**Figure 3A**) and CX3CL1 (**Figure 3C**) were noted in endothelial cells lining new blood vessels. In the stroma, CXCL16 expression was detected in CD45-expressing leukocytes (**Figure 3D**), as well as in spindle-shaped cells. In serial sections, the distribution and morphology of spindle-shaped cells expressing CXCL16 (**Figure 3B**) were similar to those of myofibroblasts expressing α-SMA (**Figure 2E**). The stromal cells expressing CX3CL1 were leukocytes co-expressing CD45 (**Figure 3E**).

We also provided a quantitative assessment of the qualitative immunohistochemistry stainings. The mean number of blood vessels expressing CXCL16 (56.3 ± 32.1) was significantly higher than the mean number of blood vessels expressing CX3CL1 (29.0 ± 20.4) (p<0.001; paired sample t-test). Similarly, the mean number of stromal cells expressing CXCL16 (83.3 ± 47.8) was significantly higher than the mean number of stromal cells expressing CX3CL1 (15.5 ± 19.7) (p<0.001; paired sample t-test).

**Figure 4:**
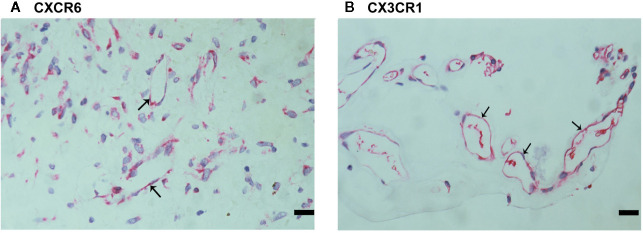
Characterization of CXCR6- and CX3CR1-expressing cells in proliferative diabetic retinopathy epiretinal fibrovascular membranes. Immunoreactivities for CXCR6 **(A)** and CX3CR1 **(B)** were detected in vascular endothelial cells. Immunoreactivity for CXCR6 was also detected in stromal spindle-shaped cells **(A)** (scale bar, 10 μm).

Since CXCL16 and CX3CL1 activities required binding to their sole receptors CXCR6 and CX3CR1, respectively (22), we also evaluated CXCR6 and CX3CR1 expression in epiretinal fibrovascular membranes from patients with PDR. CXCR6 (**Figure 4A**) and CX3CR1 (**Figure 4B**) immunoreactivities were detected in vascular endothelial cells. Immunoreactivity for CXCR6 was also noted in stromal cells. CXCR6-expressing stromal cells were spindle-shaped cells (**Figure 4A**) resembling α-SMA-expressing myofibroblasts (**Figure 2E**). These results implied that both the ligands and the receptors were locally co-expressed and might result in cell adhesion and other biological effects.

### Expression of ADAM10 and ADAM17 in Epiretinal Fibrovascular Membranes From Patients With PDR

**Figure 5:**
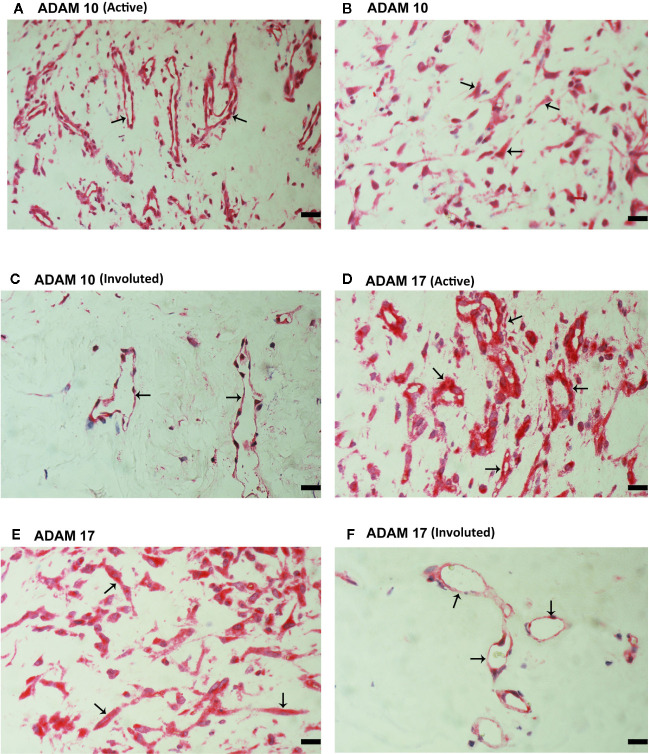
Characterization of ADAM10- and ADAM17-expressing cells in proliferative diabetic retinopathy epiretinal fibrovascular membranes. Immunoreactivities for ADAM10 **(A, C)** and ADAM17 **(D, F)** were detected in vascular endothelial cells (arrows) in membranes from patients with active neovascularization **(A, D)** and in membranes from patients with inactive involuted disease **(C, F)**. Immunoreactivities for ADAM10 **(B)** and ADAM17 **(E)** were also detected in stromal spindle-shaped cells (arrows) (scale bar, 10 μm).

**Figure 6:**
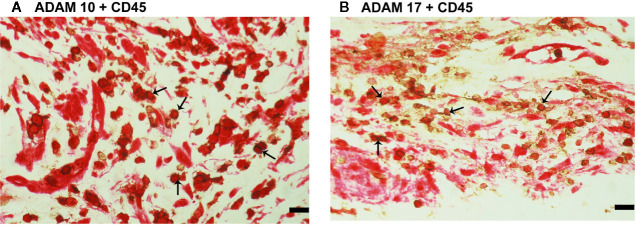
Characterization of ADAM10- and ADAM17-expressing cells in proliferative diabetic retinopathy epiretinal fibrovascular membranes. Double immunohistochemistry for CD45 (brown) and ADAM10 (red) **(A)** or ADAM17 (red) **(B)** showed co-expression in leukocytes (arrows). No counterstain to visualize the cell nuclei was applied in panels **(A, B)** (scale bar, 10 μm).

CXCL16 and CX3CL1 are unique chemokines being shed from their membrane forms by ADAM10 and ADAM17 (26–31). To determine whether these interactions may take place in the ocular microenvironment, we studied these metalloproteases by immunolocalisation. Immunoreactivities for ADAM10 (**Figures 5A–C**) and ADAM17 (**Figures 5D–F**) were observed in all membranes and were noted in vascular endothelial cells and stromal cells. Representative examples of stainings in active (**Figures 5A, B, D, E**) and involuted (**Figures 5C, F**) membranes are shown. Stromal cells were spindle-shaped cells expressing α-SMA (**Figures 5B, E**) and leukocytes co-expressing CD45 (**Figures 6A, B**).

### Correlations Between Expression Levels of Transmembrane Chemokines and Processing ADAMs and Angiogenic Activity in Epiretinal Fibrovascular Membranes From Patients With PDR

Significant positive correlations (Pearson correlation coefficients) were detected between the numbers of blood vessels expressing CD31, reflecting the angiogenic activity of PDR epiretinal fibrovascular membranes, and the number of blood vessels expressing CXCL16, CX3CL1, CXCR6, CX3CR1, ADAM10 and ADAM17. In addition, significant positive correlations were found between the numbers of blood vessels expressing CD31 and the numbers of stromal cells immunoreactive for CXCL16, CXCR6, ADAM10 and ADAM17 (**Table 2**).

**Table 2:** Correlations (Pearson correlation coefficient) between microvessel density (MVD) and the numbers of immunoreactive vessels and stromal cells in epiretinal fibrovascular membranes from patients with proliferative diabetic retinopathy.

	Variable	r	p-value
MVD	• Blood vessels expressing CXCL16	0.530	0.024*
	• Stromal cells expressing CXCL16	0.661	0.003*
	• Blood vessels expressing CX3CL1	0.510	0.031*
	• Stromal cells expressing CX3CL1	0.041	0.870
	• Blood vessels expressing CXCR6	0.581	0.014*
	• Stromal cells expressing CXCR6	0.678	0.003
	• Blood vessels expressing CX3CR1	0.616	0.006*
	• Blood vessels expressing ADAM10	0.736	0.006*
	• Stromal cells expressing ADAM10	0.753	0.005*
	• Blood vessels expressing ADAM17	0.679	0.011*
	• Stromal cells expressing ADAM17	0.565	0.044*

*Statistically significant at 5% level of significance.

### Expression of α-SMA, the Leukocyte Common Antigen CD45 and Transmembrane Chemokines and Processing ADAMs in Epiretinal Fibrocellular Membranes From Patients With PVR

**Figure 7:**
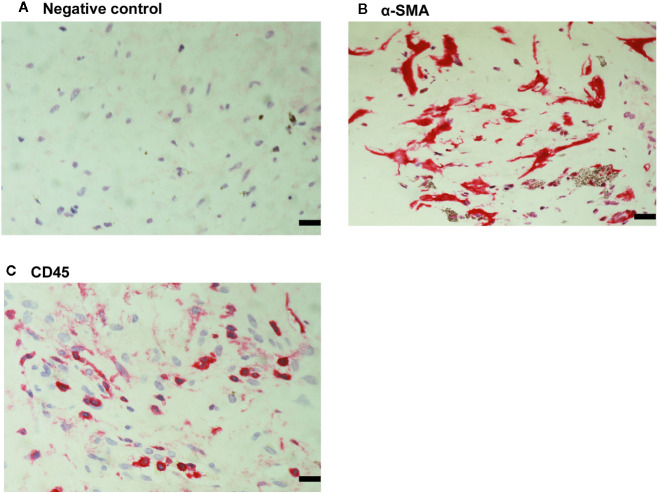
Detection of myofibroblasts and leukocytes in proliferative vitreoretinopathy epiretinal fibrocellular membranes. No staining was observed in the negative control slide **(A)**. Immunohistochemical staining for α-smooth muscle actin (α-SMA) showing immunoreactivity in spindle-shaped myofibroblasts **(B)**. Immunohistochemical staining for CD45 showing infiltrating leukocytes **(C)** (scale bar, 10 μm).

**Figure 8:**
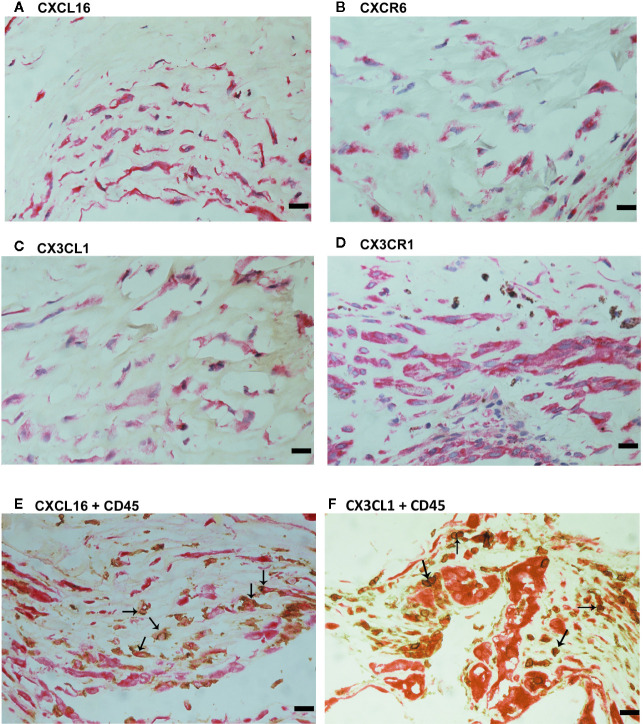
Characterization of cells expressing transmembrane chemokines and their receptors in proliferative vitreoretinopathy epiretinal fibrocellular membranes. Immunohistochemical stainings for CXCL16 **(A)**, CXCR6 **(B)**, CX3CL1 **(C)**, and CX3CR1 **(D)** showing immunoreactivities in spindle-shaped myofibroblasts. Double immunohistochemistry for CD45 (brown) and CXCL16 (red) **(E)** or CX3CL1 (red) **(F)** showed co-expression in leukocytes (arrows). No counterstain to visualize the cell nuclei was applied in panels E and F (scale bar, 10 μm).

For comparison, we used epiretinal fibrocellular membranes from patients with retinal detachment complicated by PVR. No staining was noted in the negative control slides (**Figure 7A**). All membranes showed α-SMA-expressing spindle-shaped myofibroblasts (**Figure 7B**) and CD45-expressing leukocytes (**Figure 7C**). Immunostainings for CXCL16 (**Figure 8A**), CXCR6 (**Figure 8B**), CX3CL1 (**Figure 8C**), CX3CR1 (**Figure 8D**) were detected in spindle-shaped α-SMA-expressing myofibroblasts (**Figure 7B**). In addition, double-labeling analysis showed that cells expressing CXCL16 (**Figure 8E**) and CX3CL1 (**Figure 8F**) co-expressed CD45.

**Figure 9:**
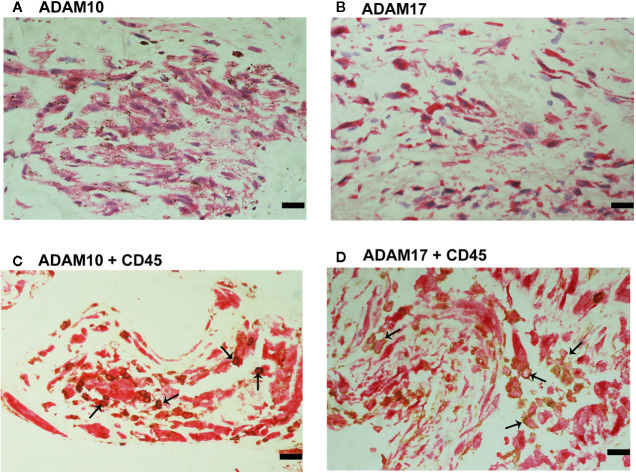
Characterization of ADAM10- and ADAM17-expressing cells in proliferative vitreoretinopathy epiretinal fibrocellular membranes. ADAM10 **(A)** and ADAM17 **(B)** immunoreactivities were detected in spindle-shaped myofibroblasts. Double immunohistochemistry for CD45 (brown) and ADAM10 (red) **(C)** or ADAM17 **(D)** demonstrated co-expression in leukocytes (arrows). No counterstain to visualize the cell nuclei was applied in panels **(C, D)** (scale bar, 10 μm).

Immunoreactivities for ADAM10 (**Figure 9A**) and ADAM17 (**Figure 9B**) were detected in spindle-shaped cells expressing the myofibroblast marker α-SMA (**Figure 7B**). In addition, double immunohistochemistry analysis demonstrated that also cells expressing ADAM10 (**Figure 9C**) and ADAM17 (**Figure 9D**) co-expressed CD45.

### CXCL16 Induces Upregulation of VEGF, Phospho-ERK1/2, and the p65 Subunit of NF-κB in Retinal Müller Glial Cells

**Figure 10:**
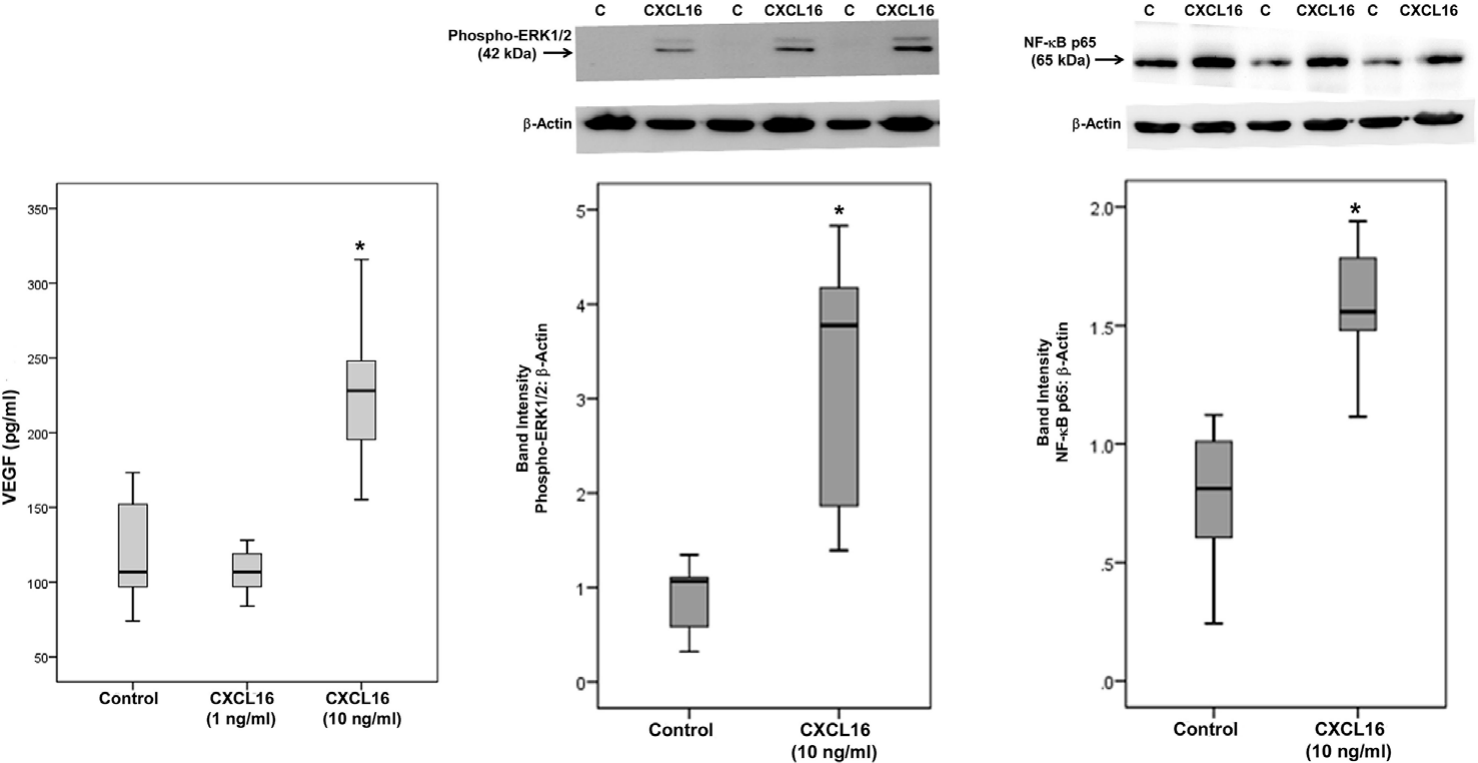
CXCL16 induces vascular endothelial growth factor (VEGF) expression and activates ERK1/2 and NF-κB pathways in Müller cells. Müller cells were left untreated or treated with CXCL16 for 24 h. Levels of VEGF were quantified in the culture media by enzyme-linked immunosorbent assay (ELISA). Protein expression of phospho-ERK1/2 and the p65 subunit of NF-κB in the cell lysates was determined by Western blot analysis (representative Western blots are depicted on top of the graphs). The box plots (median and interquartile range) show results from three different experiments performed in triplicate. (*p < 0.05; Mann-Whitney test).

As the levels of CXCL16 in the intraocular microenvironment of patients with PDR were several orders of magnitude higher than the levels of CX3CL1 (**Table 1**), we focused on CXCL16 in subsequent *in vitro* studies. We addressed the questions (i) what is the local effect of high CXCL16, (ii) which factor(s) may influence the relatively high production of CXCL16 and (iii) whether two functions of CXCL16, namely cell adhesion and angiogenesis, may occur locally. To confirm the significant positive correlation between vitreous fluid levels of CXCL16 and VEGF, we performed induction experiments on retinal Müller glial cells with CXCL16 as an inducer of VEGF production. We found that at 10 ng/ml, CXCL16 significantly increased the levels of VEGF in the culture medium after an incubation of 24 h. However, stimulation with 1 ng/ml CXCL16 did not affect the expression of VEGF as compared to untreated control (**Figure 10**). Western blot analysis demonstrated that treatment of Müller cells with CXCL16 (10 ng/ml) induced significant upregulation of the protein levels of phospho-ERK1/2 and the p65 subunit of NF-κB (**Figure 10**).

### The Pro-inflammatory Cytokine TNF-α Induces Upregulation of Soluble CXCL16 and ADAM17 in Retinal Müller Glial Cells

**Figure 11:**
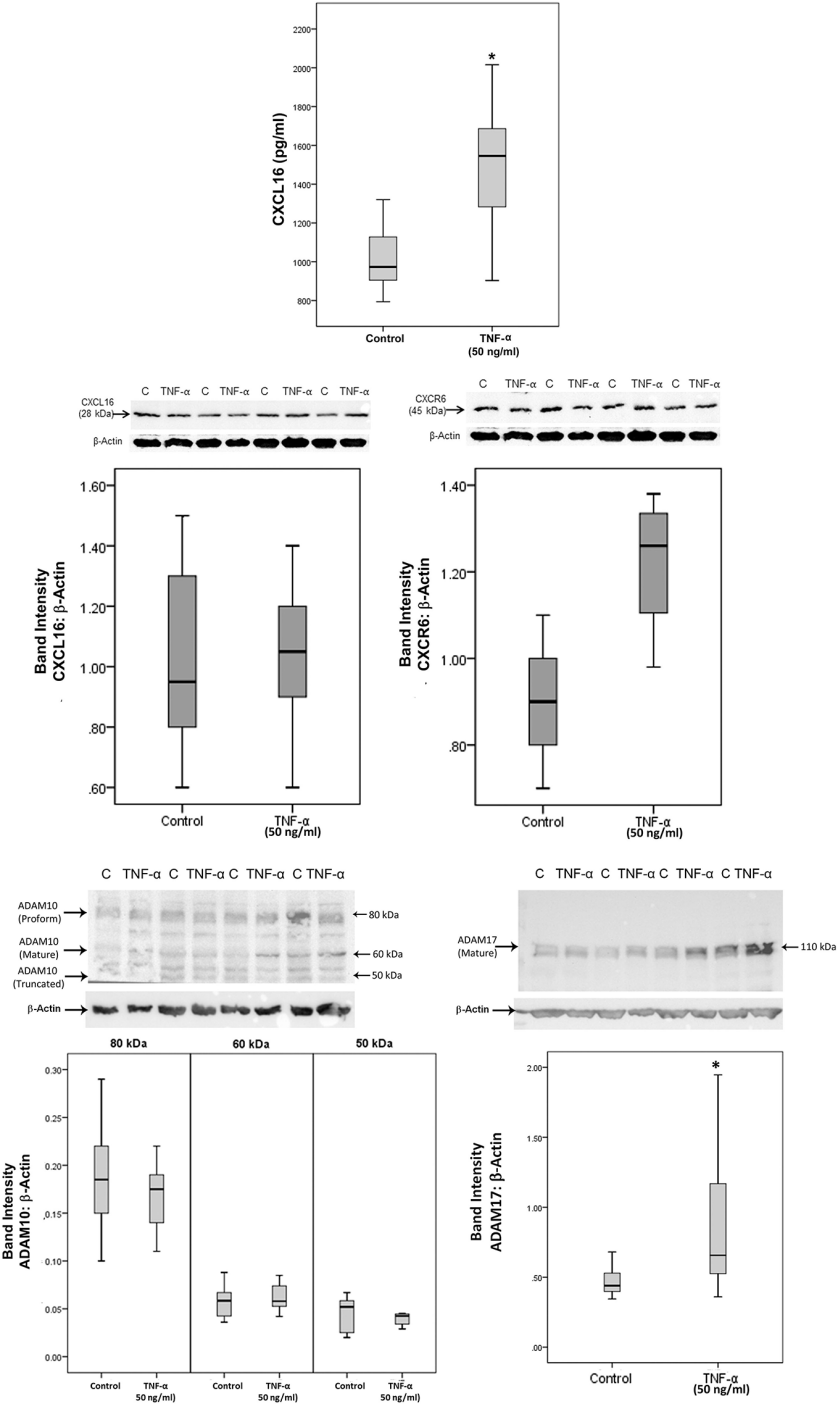
The proinflammatory cytokine tumor necrosis factor-alpha (TNF-α) induces the expression of CXCL16 and ADAM17 in Müller cells. Müller cells were left untreated or treated with TNF-α (50 ng/ml) for 24 h. Levels of CXCL16 were quantified in the culture media by ELISA. Protein expression of CXCL16, CXCR6, ADAM10, and ADAM17 in cell lysates was determined by Western blot analysis. Results are expressed as median (interquartile range) from three different experiments performed in triplicate. (*p < 0.05; Mann-Whitney test).

Treatment with the pro-inflammatory cytokine TNF-α significantly increased the levels of the soluble form of CXCL16 in the culture medium as compared to untreated control (**Figure 11**). As a hypoxia mimetic agent, CoCl_2_ treatment did not affect the expression of soluble CXCL16 as compared to untreated control (data not shown). Western blot analysis of cell lysates demonstrated that treatment of Müller cells with TNF-α induced significant upregulation of ADAM17, but did not affect the expression of the transmembrane proteoform of CXCL16, and ADAM10 (**Figure 11**). CXCR6 expression showed a non-statistically significant increase (**Figure 11**).

### CXCL16, CXCR6, CX3CL1, and CX3CR1 Expression on Human Retinal Microvascular Endothelial Cells

**Figure 12:**
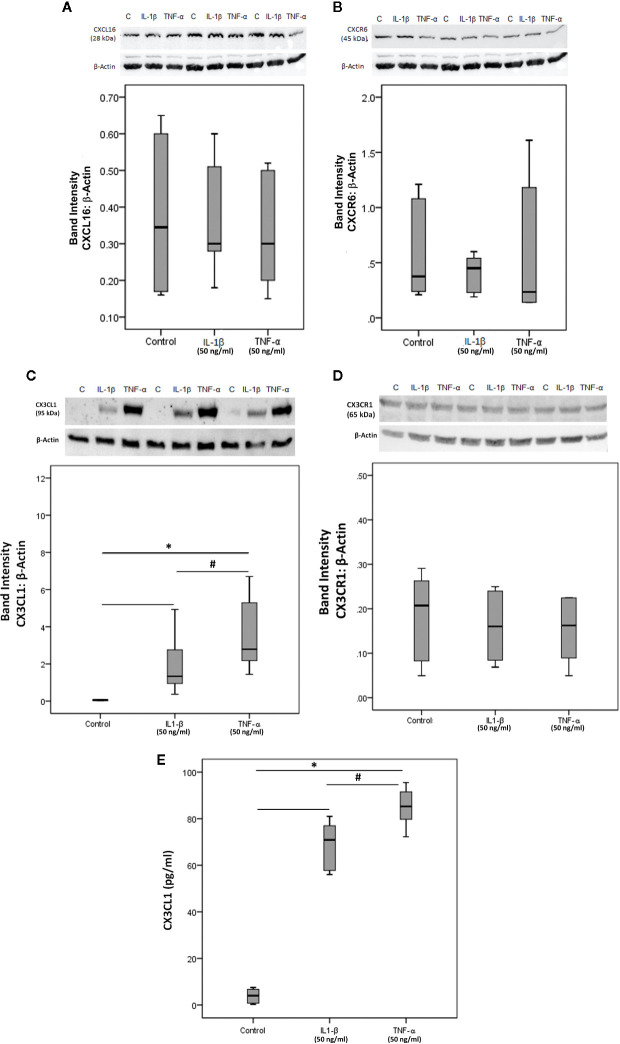
Human retinal microvascular endothelial cells (HRMECs) express CXCL16, CXCR6, CX3CL1, and CX3CR1. HRMECs were left untreated or treated with interleukin-1 beta (IL-1β) (50 ng/ml) or tumor necrosis factor-alpha (TNF-α) (50 ng/ml) for 24 h. Protein expression of CXCL16 **(A)**, CXCR6 **(B)**, CX3CL1 **(C),** and CX3CR1 **(D)** in cell lysates was determined by Western blot analysis (representative Western blots are depicted on top of the graphs). The same loading control (β-actin) was used for quantitation of the relative band intensity of both CXCL16 and CXCR6. Levels of CX3CL1 were quantified in the culture media by ELISA **(E)**. The box plots (median and interquartile range) show results from three different experiments performed in triplicate. Kruskal-Wallis test and Mann-Whitney tests were used for comparisons between three groups and two groups, respectively. *P < 0.05 compared with values obtained from untreated cells. ^#^p < 0.05 compared with IL-1β-treated cells.

To confirm the observed expression of CXCL16, CXCR6, CX3CL1 and CX3CR1 by endothelial cells in epiretinal fibrovascular membranes from patients with PDR, we performed *in vitro* experiments on HRMECs. We showed by Western blot analysis that HRMECs express CXCL16 (**Figure 12A**), CXCR6 (**Figure 12B**), CX3CL1 (**Figure 12C**) and CX3CR1 (**Figure 12D**). Exposure of HRMECs to IL-1β or TNF-α did not affect the expression of the membrane-bound proteoform of CXCL16 (**Figure 12A**), CXCR6 (**Figure 12B**) and CX3CR1 (**Figure 12D**). In addition, ELISA analysis demonstrated that the soluble form of CXCL16 was not detected in the culture medium of untreated or treated cells. In contrast, treatment with IL-1β or TNF-α induced significant upregulation of the transmembrane proteoform of CX3CL1 in the cell lysates (**Figure 12C**) and the soluble form of CX3CL1 in the culture medium (**Figure 12E**).

### CXCL16 Induces Leukocyte Adhesion to Human Retinal Microvascular Endothelial Cells and Upregulation of the Cell Adhesion Molecule ICAM-1

**Figure 13:**
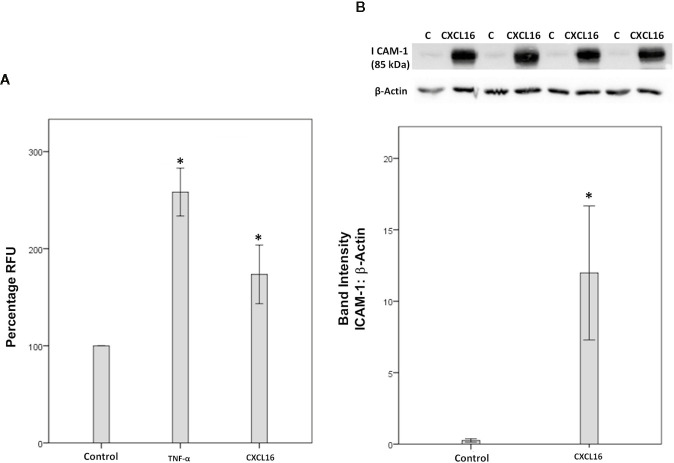
CXCL16 induces leukocyte adhesion to human retinal microvascular endothelial cells (HRMECs). Adhesion of fluorescently-labeled monocytic cells to a HRMEC monolayer treated with tumor necrosis factor‐α (TNF‐α) (50 ng/ml) or CXCL16 (50 ng/ml) was assessed. Bar graphs represent three independent experiments (control: 11 wells, TNF‐α: 11 wells and CXCL16: 11 wells). RFU, relative fluorescent unit. One-way ANOVA and independent t-tests were used for comparisons between the three and two groups, respectively **(A)**. HRMECs were left untreated or treated with CXCL16 (50 ng/ml) for 24 h. Protein expression of intercellular adhesion molecule-1 (ICAM-1) in cell lysates was determined by Western blot analysis **(B)**. Results are expressed as mean ± standard deviation from three different experiments performed in triplicate. (*p < 0.05; independent t-test).

Since we demonstrated that HRMEC express CXCR6, both by Western blot analysis (**Figure 12B**) and immunocytochemistry (data not shown), we next investigated how the CXCL16/CXCR6 axis could be involved in the development of diabetic retinopathy. Enhanced adhesion of circulating leukocytes to the vascular endothelium is a hallmark feature of diabetic retinopathy (13). Classically, this adhesion is executed by interactions between leukocyte functional antigen-1 and ICAM-1, both molecules being inducible by TNF-α. Therefore, TNF-α (50 ng/ml) was used as a positive control. Similar to the TNF-α effect, we found that treatment of HRMECs with 50 ng/ml CXCL16 significantly upregulated the adherence of leukocytes to HRMECs (**Figure 13A**). However, treatment with 10 ng/ml CXCL16 did not affect the adherence of leukocytes to HRMECs (data not shown). With the use of Western blot analysis, we demonstrated that treatment of HRMECs with CXCL16 (50 ng/ml) for 24 h resulted in increased expression of ICAM-1 compared with the control medium (**Figure 13B**). However, treatment with 10 ng/ml CXCL16 did not affect the expression of ICAM-1 as compared to untreated control (data not shown).

### CXCL16 Induces Proliferation and Upregulation of Phospho-ERK1/2 in Human Retinal Microvascular Endothelial Cells and Formation of Sprouts From Human Retinal Microvascular Endothelial Cell Spheroids

**Figure 14:**
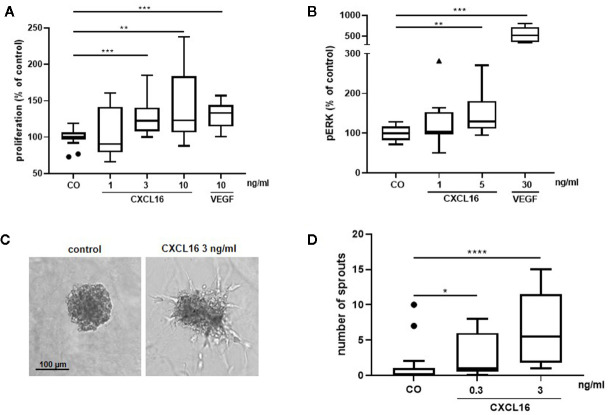
CXCL16 induces proliferation, phosphorylation of ERK1/2 and angiogenic sprouting in human retinal microvascular endothelial cells (HRMECs). HRMECs were stimulated with control medium alone or control medium with varying concentrations of CXCL16 (1 to 10 ng/ml) or with 10 ng/ml VEGF as a positive control. The number of metabolically active cells was quantified after 48 h using the ATPlite assay and expressed relative to control. (n=2 to 4, in quadruplicate) **(A)**. The amount of phospho-ERK1/2 in HRMECs stimulated with CXCL16 (1 or 5 ng/ml) or VEGF (30 ng/ml) for 15 min was measured through enzyme-linked immunosorbent assay (ELISA). Relative phospho-ERK1/2 levels compared to unstimulated control are shown. (n=4, in duplicate or triplicate) **(B)**; HRMEC spheroids were treated with medium (control), 0.3 or 3 ng/ml of CXCL16. After 14 h of incubation, spheroid sprouting was assessed in two independent experiments. Representative images of a control spheroid and a spheroid stimulated with 3 ng/ml CXCL16 are shown **(C)**. For each condition (control, n=17; 0.3 ng/ml, n=17; 3 ng/ml, n=14), the number of sprouts per spheroid was counted **(D)**. All results are expressed as median (interquartile range) ((*p < 0.05, **p < 0.01,***p < 0.001, ****p < 0.0001, Mann-Whitney test).

To further explore the angiogenic effects of CXCL16, *in vitro* experiments with HRMECs were performed. As a positive control, cells stimulated with VEGF were included in every assay. HRMECs stimulated with 3 or 10 ng/ml CXCL16 for 48 h showed a significantly higher proliferative index compared to control cells (**Figure 14A**). As activation of the ERK signaling pathway is an important driver of angiogenesis, phospho-ERK1/2 content was also measured in response to CXCL16. HRMECs stimulated with 5 ng/ml CXCL16 for 15 min exhibited a higher level of phospho-ERK1/2 compared to cells stimulated with control medium alone (**Figure 14B**). HRMEC sprouting was observed after 14-h treatment of spheroids with CXCL16. The highest increase in the number of sprouts was seen at a dose of 3 ng/ml CXCL16 (**Figures 14C, D**).

### Effect of Intravitreal Administration of CXCL16 on Retinal Expression of the p65 Subunit of NF-κB, Phospho-ERK1/2, ICAM-1, and VEGF

**Figure 15:**
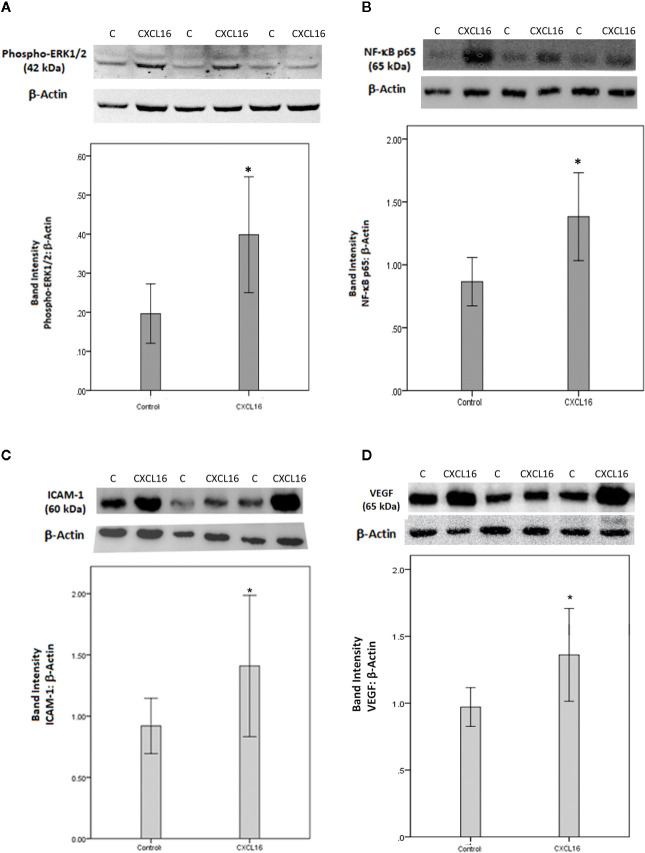
Western blot analysis of rat retinas. Rats received intravitreal injection of human CXCL16 (5 ng/5 μl) in the right eye and phosphate-buffered saline (PBS) in the left eye. The expression levels of phospho-ERK1/2 **(A)**, the p65 subunit of NF-κB **(B)**, intercellular adhesion molecule-1 (ICAM-1) **(C)** and vascular endothelial growth factor (VEGF) **(D)** were assessed by Western blot analysis after 24 h **(A, B)** or 4 days **(C, D)**. Results are expressed as mean ± standard deviation. *p < 0.05 (independent t-test) compared to the values obtained from PBS-injected eyes.

Finally, to integrate our findings and to translate the *in vitro* data on CXCL16 to *in vivo* relevance, we studied the effects of intravitreal injection of CXCL16 in rat eyes in comparison to those of sham-injections in contralateral eyes. We evaluated changes in several inflammatory mediators one day and 4 days after injection to be able to distinguish early and later processes. Western blot analysis of homogenized retinal tissue demonstrated that intravitreal injection of CXCL16 induced significant upregulation of the protein levels of phospho-ERK1/2 (**Figure 15A**) and the p65 subunit of NF-κB (**Figure 15B**) (n=9) after one day of intravitreal administration compared to values obtained from the contralateral eye that received PBS alone (n=9). After 4 days of intravitreal injection, there was significant upregulation of the protein levels of the p65 subunit of NF-κB (data not shown), ICAM-1 (**Figure 15C**) and VEGF (**Figure 15D**) (n=11) compared to the values obtained from the contralateral eye that received PBS alone (n=12). However, the expression of phospho-ERK1/2 was not affected at the 4 day timepoint (data not shown), indicating that this signaling pathway is switched on early, but temporarily, whereas activation of NF-κB was more prolonged in time. The rat streptozotocin-induced diabetes model is useful to study hyperglycemia-induced early retinal changes such as breakdown of blood-retinal barrier and inflammation. It is noteworthy that the animals in this model do not develop PDR resulting in retinal angiogenesis and epiretinal fibrovascular proliferation as in humans.

## Discussion

In the present study, we add novel data on the relative expression of the transmembrane chemokines CXCL16 and CX3CL1 in the ocular microenvironment of patients with PDR. Among the transmembrane chemokines, the expression levels of CXCL16 were significantly higher than the expression levels of CX3CL1. Our analysis also showed that the levels of CXCL16 in the vitreous fluid of patients with PDR were 417-fold higher than those of CX3CL1. Our findings suggest a predominant role for CXCL16 over CX3CL1 as a proinflammatory and proangiogenic chemokine in PDR. Similarly, a previous study detected very low levels of CX3CL1 in the vitreous fluid of patients with epiretinal membranes and macular holes that were lower than the CXCL16 levels (52). We detected for the first time the co-expression of CXCL16 and its receptor CXCR6 in endothelial cells, leukocytes and myofibroblasts in epiretinal fibrovascular membranes from patients with PDR. In addition, CXCL16 and CXCR6 were prominently expressed by cultured HRMECs. These results are in line with those of previous studies that demonstrated CXCL16 expression by tumor-associated endothelial cells, leukocytes and fibroblasts (53) and CXCR6 expression by human dermal microvascular endothelial cells (40).

Responses involving expression of CXCL16 and CXCR6 in the presence of proinflammatory cytokines may depend on cell type. TNF-α treatment upregulated the expression of CXCL16 in ectopic endometrial stromal cells (54), keratinocytes (55), macrophages (56), prostate epithelial cells (57), lymph node fibroblastic reticular cells (58) and gingival fibroblasts (59). Similarly, IL-1β treatment induced CXCL16 expression in prostate epithelial cells (57), gingival fibroblasts (59), peripheral blood mononuclear cells, umbilical vein endothelial cells (60), and annulus fibrosus cells (61). Additionally, TNF-α treatment enhanced CXCR6 expression in gingival fibroblasts (62) and prostate epithelial cells (57) and IL-1β treatment induced CXCR6 expression in prostate epithelial cells (57). In contrast, stimulation of rheumatoid arthritis synovial tissue fibroblasts (63), bronchial epithelial cells (64), smooth mushle cells (27, 65), umbilical vein endothelial cells (27) and annulus fibrosus cells (61) by TNF-α did not influence CXCL16 expression. Additionally, IL-1β treatment did not upregulate the production of CXCL16 by rheumatoid arthritis synovial tissue fibroblasts (63). Similarly, TNF-α had no effect on CXCR6 expression in ectopic endometrial stromal cells (54) and IL-1β did not change CXCR6 levels in gingival fibroblasts (62). We also found no increase of CXCL16 and CXCR6 when we exposed HRMECs to TNF-α or IL-1β prior to Western blot analysis. In contrast, in response to the same stimuli, HRMECs produced significantly elevated levels of CX3CL1. Our findings are in agreement with previous reports that demonstrated CX3CL1, but not CXCL16 induction by TNF-α in rheumatoid arthritis synovial tissue fibroblasts (63, 66).

In addition to its role in leukocyte recruitment and inflammation, the CXCL16/CXCR6 signaling pathway has been shown to influence angiogenesis. Several studies demonstrated that CXCL16 is a novel angiogenic factor. CXCL16 promoted human umbilical vein endothelial cell (38, 39) and human dermal microvascular endothelial cell (40) migration, proliferation and tube formation *in vitro*, key steps in early angiogenesis. It has been shown that CXCL16 is an important mediator of angiogenesis in rheumatoid arthritis (40). Overexpression of CXCR6 on implanted prostate cancer cell lines resulted in increased blood vessel formation in mice (41). Downregulation of CXCR6 in subcutaneously implanted hepatocellular carcinoma xenografts in mice, led to suppression of inflammatory infiltration and angiogenesis *via* inhibition of proinflammatory cytokine production (42). In the present study, we demonstrated that the vitreous levels of CXCL16 were significantly higher in eyes with active neovascularization in comparison with eyes with involuted disease. In addition, there were significant positive correlations between the expression levels of the CXCL16/CXCR6 chemokine axis and the degree of angiogenic activity in PDR epiretinal fibrovascular membranes. Furthermore, we demonstrated that CXCL16 induced HRMEC proliferation, formation of sprouts from HRMEC spheroids and phosphorylation of ERK1/2 *in vitro.* Taken together, these findings suggest that the CXCL16/CXCR6 chemokine pathway might contribute to the pathogenesis of pathological angiogenesis associated with PDR.

In the current study, our analysis demonstrated a significant positive correlation between the vitreous fluid levels of CXCL16 and those of the angiogenic biomarker VEGF, a key angiogenic factor in PDR (10, 11). The co-expression of CXCL16 and VEGF in the ocular microenvironment of patients with PDR suggests a crosstalk between these factors in the pathogenesis of PDR angiogenesis and that co-expression of these factors is mechanistically interrelated. To corroborate the findings at the cellular level, we demonstrated for the first time the capability of CXCL16 to target Müller cells and to induce upregulation of the p65 subunit of NF-κB and phospho-ERK1/2 and synthesis and secretion of VEGF. Müller cells, a major source of VEGF secretion, contribute to the development of pathological retinal neovascularization (67). In addition, intravitreal injection of CXCL16 induced upregulation of the p65 subunit of NF-κB, phospho-ERK1/2 and VEGF in the rat retinas. These results are in line with previous studies that demonstrated the capacity of CXCL16 to induce VEGF in human umbilical vein endothelial cells (38, 39) and tumor cells (41). In addition, CXCL16 binding to its receptor CXCR6 induced upregulation of phospho-ERK1/2 in human umbilical vein endothelial cells (38, 39) and activated the pro-inflammatory transcription factor NF-κB signaling pathway (68). These results suggest that one possible mechanism of CXCL16-induced pathological neovascularization in PDR is related to the upregulation of VEGF. Furthermore, we showed that treatment of Müller cells with the proinflammatory cytokine TNF-α enhanced the levels of soluble CXCL16 in the culture medium. These findings are in agreement with previous reports that demonstrated CXCL16 induction by TNF-α in several types of cells (54–59).

In the present study, our immunohistochemical analysis demonstrated CXCL16 and CXCR6 localization in α-SMA-expressing myofibroblasts, the key cellular mediator of fibrosis (6), in epiretinal membranes from patients with PDR and PVR. These findings are consistent with previous studies documenting CXCL16 localization in cancer-associated fibroblasts (53, 69). In addition to its role in inflammation and angiogenesis, CXCL16 activity may represent an important determinant in the pathogenesis of organ fibrosis. In a mouse model of renal fibrosis it was demonstrated that CXCL16 gene expression was induced in the kidney and that CXCL16 plays a pivotal role in the pathogenesis of renal fibrosis. Targeted disruption of CXCL16 prevented the development of renal fibrosis by suppressing the recruitment of CXCR6-expressing bone marrow-derived fibroblast precursors, macrophages and T cells into the kidney and myofibroblast formation (70).

ADAMs are a family of membrane-associated proteinases that mediate proteolytic ectodomain shedding of membrane-associated proteins. The proteolytic release of extracellular domains of transmembrane proteins is an important regulator of diverse biological processes, including inflammation and pathological neovascularization (71). To our knowledge, this is the first report to investigate the expression of ADAM10 and ADAM17 in the ocular microenvironment of patients with PDR. ADAM10 and ADAM17 levels were significantly elevated in the vitreous fluid from patients with PDR. Furthermore, the levels were significantly higher in patients with active neovascularization in comparison with patients with quiescent disease. Moreover, our analysis demonstrated significant positive correlations between the levels of angiogenic activity and the levels of ADAM10 and ADAM17 expression in PDR epiretinal fibrovascular membranes. Altogether, these observations are consistent with those of previous cancer studies that demonstrated a role for ADAM10 (72, 73) and ADAM17 (74–77) in pathological angiogenesis. In the present study, we demonstrated that ADAM10 levels were significantly higher than ADAM17 levels in the vitreous fluid. It was demonstrated that ADAM10 is the predominant protease responsible for the largest proportion of constitutive shedding of CXCL16 (26–28) and CX3CL1 (28, 29). In contrast, the phorbol-12-myristate-13-acetate (PMA)-induced shedding of CXCL16 (28) and CX3CL1 (28, 30, 31) is attributed to the induced activity of ADAM17.

In the current study, we detected simultaneous expression of ADAM10, ADAM17 and CXCL16 in the vitreous fluid from patients with PDR. There were significant positive correlations between the levels of CXCL16 and the levels of ADAM10 and ADAM17. In addition, we detected the co-expression of ADAM10, ADAM17 and CXCL16 in endothelial cells, leukocytes and myofibroblasts in epiretinal fibrovascular membranes from patients with PDR. These findings are in accordance with previous studies that demonstrated active involvement of ADAM10 and ADAM17 in the proteolytic release of CXCL16 from the cell surface generating the soluble molecule (26–28). Thus, vitreous levels of specific CXCL16 proteoforms might reflect ADAM activity in the ocular microenvironment of patients with PDR. The co-expression of ADAM10, ADAM17 and CXCL16 suggests a crosstalk between these factors in the pathogenesis of inflammation and angiogenesis in PDR. Transmembrane CXCL16 expression on the surface of endothelial cells functions as adhesion molecule that might promote adhesion and firm arrest of leukocytes expressing CXCR6. In the present study, we demonstrated that exogenous CXCL16 activated endothelial cells to upregulate the adhesion molecule ICAM-1 and enhanced the binding of leukocytes to CXCL16-activated HRMECs. In addition, CXCL16 expressed on the endothelium triggers platelet activation and adhesion to the endothelium *via* its receptor, suggesting a role for CXCL16 in vascular inflammation and thrombo-occlusive diseases (78). Proteolytic shedding of CXCL16 by ADAM10 and ADAM17 would lead to generation of soluble chemokine and the generation of a chemotactic gradient that could further direct the transmigrating leukocytes from the endothelium into the underlying tissue. Thus, proteolytic cleavage of CXCL16 appears to be essential for its switch from an adhesive to a chemoattactive function and may be regarded as prerequisite for its proinflammatory and proangiogenic effects.

In conclusion, the present study demonstrated that the chemokine axis CXCL16/CXCR6 and the processing metalloproteinases ADAM10 and ADAM17 are co-expressed in the ocular microenvironment of patients with PDR. These findings suggest that this signaling axis serves a role in the initiation and progression of PDR. Intervention studies would provide conclusive evidence and will be part of future studies. Such experiment, together with our data on the role of CXCL16 in angiogenesis associated with PDR would support the possible use of CXCL16/CXCR6 antagonists in PDR patients to block inflammatory angiogenesis associated with PDR.

## Data Availability Statement

The raw data supporting the conclusions of this article will be made available by the authors, without undue reservation.

## Ethics Statement

The study was conducted according to the tenets of the Declaration of Helsinki. Before undergoing vitrectomy, all patients signed a preoperative informed written consent and approved the use of the excised epiretinal membranes and aspirated vitreous fluid for further analysis and clinical research. The Research Centre and Institutional Review Board of the College of Medicine, King Saud University, approved the study design and protocol. All procedures with animals were performed in accordance with the Association for Research in Vision and Ophthalmology (ARVO) statement for use of animals in ophthalmic and vision research and were approved by the institutional animal care and use committee of the College of Pharmacy, King Saud University.

## Author Contributions

AMA designed the manuscript, supplied funding, interpreted the data, and wrote the manuscript. MN, AA, ADZ, MS, MB, and EA performed experiments and interpreted the data. PG analyzed the data. GDH designed, supervised, and interpreted IHC stainings. JVD designed experiments. GO provided funding and designed experiments. SS provided funding, designed experiments, interpreted the data and wrote the manuscript. All authors contributed to the article and approved the submitted version.

## Funding

This work was supported by King Saud University through Vice Deanship of Research Chair, Dr. Nasser Al-Rashid Research Chair in Ophthalmology (AMA). Research in the Rega Institute at KU Leuven was supported by C1 funding (C16/17/010 KU Leuven) and the Research Foundation of Flanders (FWO-Vlaanderen G0A3820N, G0A5716N, and G0A7516N).

## Conflict of Interest

The authors declare that the research was conducted in the absence of any commercial or financial relationships that could be construed as a potential conflict of interest.
